# Radiomics and Hybrid Models Based on Machine Learning to Predict Levodopa-Induced Dyskinesia of Parkinson’s Disease in the First 6 Years of Levodopa Treatment

**DOI:** 10.3390/diagnostics13152511

**Published:** 2023-07-27

**Authors:** Yang Luo, Huiqin Chen, Mingzhen Gui

**Affiliations:** 1 Department of Neurology, Xiangya Hospital, Central South University, Changsha 410083, China; luoyang2025@126.com; 2 Department of Radiology, Xiangya Hospital, Central South University, Changsha 410083, China; chenhuiqin789@126.com; 3 School of Automation, Central South University, Changsha 410083, China

**Keywords:** Parkinson’s disease, dyskinesias, magnetic resonance imaging, machine learning, levodopa

## Abstract

Background: Current research on the prediction of movement complications associated with levodopa therapy in Parkinson’s disease (PD) is limited. levodopa-induced dyskinesia (LID) is a movement complication that seriously affects the life quality of PD patients. One-third of PD patients develop LID within 1 to 6 years of levodopa treatment. This study aimed to construct models based on radiomics and machine learning to predict early LID in PD. Methods: We extracted radiomics features from the T1-weighted MRI obtained in the baseline of 49 PD control and 54 PD with LID in the first 6 years of levodopa therapy. Six brain regions related to the onset of PD were segmented as regions of interest (ROIs). The least absolute shrinkage and selection operator (LASSO) was used for feature selection. Using the machine learning methods of support vector machine (SVM), random forest (RF), and AdaBoost, we constructed radiomics models and hybrid models. The hybrid models combined the radiomics features and the Unified Parkinson’s Disease Rating Scale part III (UPDRS III) total score. The five-fold cross-validation was performed and repeated 20 times to validate the stability of the classifiers. We used sensitivity, specificity, accuracy, receiver operating characteristic (ROC) curves, and area under the ROC curve (AUC) for model validation. Results: We selected 33 out of 6138 radiomics features. In the testing set of the radiomics model, the AUC values of the SVM, RF, and AdaBoost classifiers were 0.905, 0.808, and 0.778, respectively, and the accuracies were 0.839, 0.742, and 0.710. The hybrid models had better prediction performance. In the testing set, the AUC values of SVM, RF, and AdaBoost classifiers were 0.958, 0.861, and 0.832, respectively, and the accuracies were 0.903, 0.806, and 0.774. Conclusions: Our results indicate that T1-weighted MRI is valuable in predicting early LID in PD. This work demonstrates that the combination of radiomics features and clinical features has good potential and value for identifying early LID in PD.

## 1. Introduction

Parkinson’s disease (PD) is a common neurodegenerative disease with an incidence second to that of Alzheimer’s disease [1]. The main pathological features of PD are the loss of dopaminergic neurons in the substantia nigra (SN) and the abnormal deposition of a-synuclein in brain tissue [2]. With the progression of the disease, PD has a series of motor symptoms and non-motor symptoms [3], among which motor symptoms mainly include bradykinesia, dystonia, resting tremor, and ataxia. Non-motor symptoms include rapid eye movement sleep disorder (RBD), anxiety, depression, loss of flair, and cognitive impairment. These symptoms seriously affect the life quality of PD patients. At present, there is no specific treatment to cure PD [4]. A series of drugs targets improvement in the symptoms of PD. Dopamine replacement therapy is the most effective treatment for improving motor symptoms [5]. Some studies have shown that dopamine replacement therapy at the early stage of diagnosis of Parkinson’s disease is beneficial to PD. However, dopamine replacement therapy is a double-edged sword. It has significant advantages in improving the patient’s motor symptoms, but it may also be accompanied by levodopa-induced dyskinesia (LID) and motor fluctuation symptoms [6]. As the main motor complication of levodopa, LID seriously affects the life quality of PD patients and even leads to disability [7,8]. In the first 1 to 3 years of levodopa treatment, about one in four patients develop LID, and after 4 to 6 years of dopamine replacement therapy, about one-third of patients experience LID [9]. These patients who suffer early LID are more susceptible to LID when treated with levodopa and may endure worse life quality.

Neuroimages play an important role in the diagnosis of neurodegenerative diseases [10]. They have a high diagnostic value in distinguishing idiopathic Parkinson’s disease from atypical PD and other neurodegenerative diseases with Parkinson-like symptoms, such as multiple system atrophy and progressive supranuclear palsy [11]. The rapid development of magnetic resonance imaging (MRI) in recent years has also been a positive development for the diagnosis of PD [12]. Diffusion-weighted imaging (DWI), susceptibility imaging (SWI), arterial spin labeling (ASL), and resting-state functional MRI have good diagnostic value in PD. One study constructed a model to differentiate progressive supranuclear palsy and multiple system atrophy from PD by single-tensor DWI with 90–100% sensitivity and specificity [13]. In PD, an obvious change in the SN could be observed on SWI, which was called the ‘swallowtail sign’ [14]. In addition, another study found that PD has hypoperfusion in the posterior brain tissue on the ASL, which was associated with the Montreal Cognitive Assessment (MoCA) score [15]. Imaging of positron emission tomography and presynaptic dopaminergic examination in PD showed marked reductions in vesicular monoamine transporter type 2, dopamine transporter, and L-aromatic amino acid decarboxylase [16].

Radiomics has rapidly developed in recent years. Radiomics uses data-representation algorithms to extract many radiomics features from medical images. With the method of selecting effective radiomics features as sensitive biomarkers and establishing machine learning models, radiomics can predict the progression and the prognosis of diseases and identify different diseases [17]. Radiomics has been used in mature applications in the prediction and treatment of tumors [18]. It also has the potential to diagnose and treat neurodegenerative diseases [19]. A radiomics model based on resting-state fMRI of normal controls and PD was constructed to predict PD [20]. The method of a 3D Convolutional Neural Network based on T1-weighted MRI could detect PD [21]. In another study, a radiomics model of white matter segmented from T1-weighted MRI could predict PD progression [22]. A meta-analysis showed reduced connectivity in networks in resting-state fMRI in Parkinson’s disease patients with cognitive impairment [23]. Current studies are limited in predicting motor complications of PD. Radiomics may have good potential for predicting PD complications.

LID is a serious motor complication that may occur during dopamine replacement therapy [24]. The cause of LID is still unclear. Studies have found that long-term dopamine replacement therapy may cause long-term modification of the brain, which may cause LID [25]. The pathogeny of LID is related to changes in the pathways of the cortical basal ganglia thalamic circuit, which regulates normal motor function [26]. The histological differences in the basal ganglia under normal, Parkinson’s, and levodopa-induced dyskinesia are crucial for the diagnosis and treatment of Parkinson’s disease [27]. Recent studies have reported that abnormal cholinergic signaling pathways in the striatum may play an important role in the occurrence of LID [28]. In a recent study, Su et al., reported that resting fMRI showed a higher functional connection between the SN and putamen compared to LID during drug withdrawal [29].

LID has obvious clinical heterogeneity and a complex pathogenesis [30]. The onset age of PD, the duration of PD before levodopa treatment, the gender, and the levodopa dose are risk factors of LID [31]. At present, it is hard to predict whether PD patients will develop LID in the early years of dopamine replacement treatment. Predicting the occurrence of LID by assessing risk factors is always not accurate enough. In most clinical cases, we take steps to improve symptoms of motor complications after LID has occurred. Radiomics signatures may deepen our understanding and identification of LID, which may have important implications for delaying the occurrence of LID. In this study, we built radiomics-related models with the methods of machine learning, aiming to discover effective radiomics signatures to identify LID and provide new evidence for automatic recognition of early LID.

## 2. Materials and Methods

### 2.1. Patient Information

**Figure 1:**
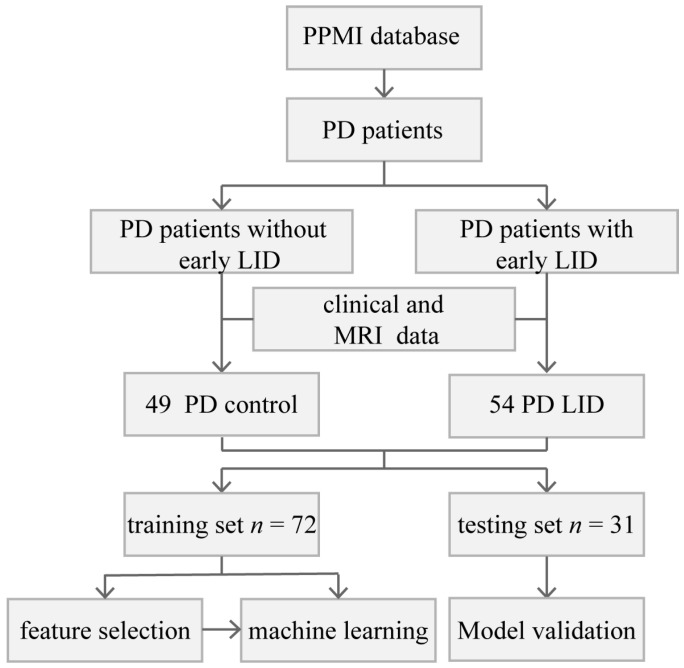
Flowchart of the current research.

Data used in this article were obtained from the Parkinson’s Progression Markers Initiative (PPMI) database (www.ppmi-info.org accessed on 1 February 2022). The database provides researchers with open-access datasets and available biobanks, which can deepen the understanding of PD [32]. Since this study is based on an open-source database, written informed consent was waived. In this study, all 103 patients met the following criteria: (I) clinically diagnosed with PD; (II) treated with dopamine replacement therapy at baseline for at least 6 years; (III) had a follow-up period for more than 6 years; and (IV) completed the MRI examination at baseline. According to the score of UPDRS part IV, patients who developed LID in the first 6 years were assigned to the PD LID group (*n* = 54). In the PD control group, patients did not develop LID in the first 6 years of levodopa treatment (*n* = 49) (Figure 1). The age, years of education, MoCA score, depression assessment, levodopa equivalent dose (LEDD), duration of illness before levodopa treatment, reflexes assessment (REF), RBD questionnaire score, and total score of the Unified Parkinson’s Disease Rating Scale part III (UPDRS III) of the two groups were tested by Levene test and ANOVA analysis, the chi-square test was performed for gender. The duration of levodopa therapy before LID in the PD LID group was also evaluated.

### 2.2. MRI Data Information

The brain MRI data of all patients were obtained from Siemens Verio 3.0T MRI machines in different clinical centers. In this study, we selected the T1-weighted MRI images obtained in the baseline. The image setting parameters were the same for each patient: repetition time = 2300 ms, echo time = 2.98 ms, slice thickness = 1 mm, the field of view (FOV) size = 256 × 240 × 256 mm, voxel size = 1 × 1 × 1 mm.

### 2.3. Image Preprocessing and ROI Segmentation

**Figure 2:**
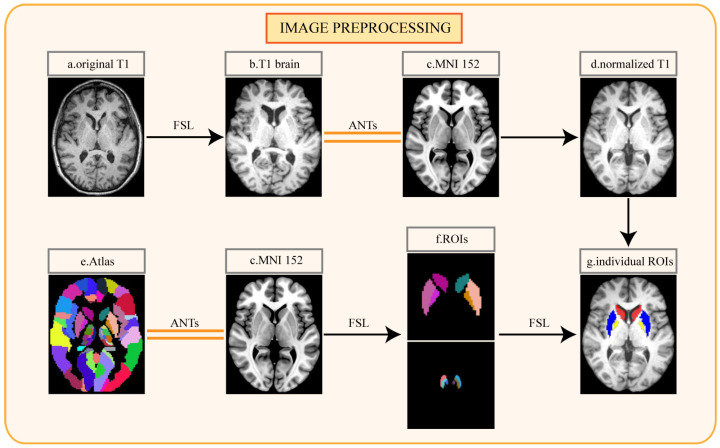
Pipeline for image preprocessing: (**a**) original T1-weighted MRI of individual patient; (**b**) T1 image after cranium removal; (**c**) Montreal Neurosciences Institute 152 (MNI152) standard space; (**d**) normalized T1 image; (**e**) automated anatomical labeling atlas 3; (**f**) the regions of interest segmented from the atlas; (**g**) the regions of interest segmented from the individual normalized image. FSL, the FMRIB Software Library; ANTs, Advanced Normalization Tools.

For further data processing, T1-weighted MRI data in DICOM format were converted to NifTI format with Python. The following image data preprocessing was performed on the T1-weighted MRI for every participant (Figure 2). The first step was the removal of non-brain tissue and bias field correction, which were performed using the FMRIB Software Library (FSL). The nonlinear normalization of each participant’s MRI to Montreal Neurosciences Institute 152 (MNI152) standard space was achieved using Advanced Normalization Tools (ANTs) [33]. Since the striatum is an important projection area of the dopaminergic neurons to the SN, the abnormalities of the striatal pathway in the SN also play an important role in the pathogenesis of PD [34]. The study of animal models showed that striatum neurons related to the globus pallidus internus were directly responsible for the development of LID [35]. These studies suggested that the activity of dopaminergic neurons in SN and the connection and projection of neural pathways in the striatum are directly or indirectly related to LID. Based on the pathological research of LID, we segmented the caudate nucleus (CAU), putamen (PUT), globus pallidus (PAL), SN pars compacta (SNpc), SN reticularis (SNpr), and ventral tegmental area (VTA) as the regions of interest (ROIs) from normalized MRI with the reference to an atlas [36]. The ROIs were segmented by FSL. The segmented ROIs were evaluated and corrected by two senior neuroradiologists with at least five years of work experience, and the two neuroradiologists were unaware of the patients’ groupings.

### 2.4. Feature Extraction and Selection 

**Figure 3:**
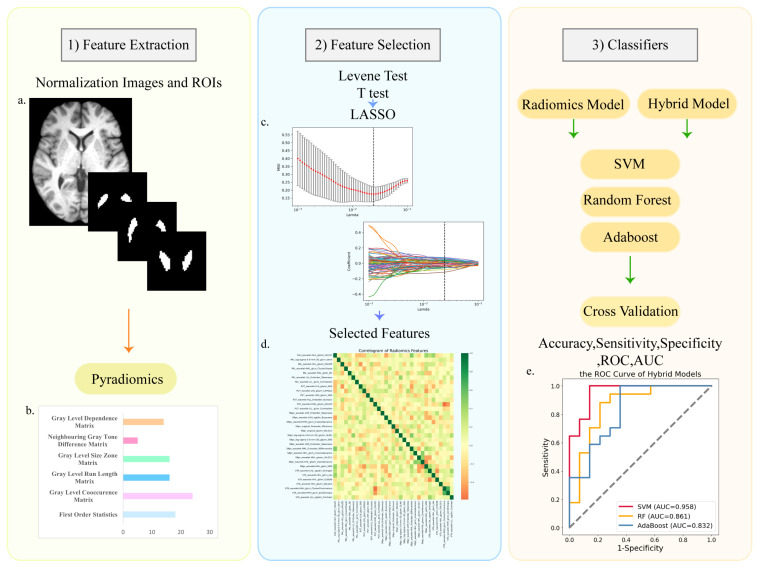
The process and main methods of constructing the radiomic models. There were three steps after image preprocessing: (1) feature extraction; (2) feature selection; (3) classifier construction. (**a**) The normalized T1 image and the masks of ROIs; (**b**) the types of radiomics features; (**c**) LASSO regression and variable filtering; (**d**) the correlation coefficient heatmap of selected features; (**e**) the ROC curve of hybrid models.

Feature extraction was based on pyradiomics, which is an open-source Python package [37], and the feature details can be obtained from the pyradiomics official website (https://pyradiomics.readthedocs.io accessed on 1 February 2022). Feature extraction was performed for 6 ROIs (CAU, PAL, PUT, SNpc, SNpr, and VTA). We extracted the original and filtered data using LoG and wavelet filters. We extracted 1023 radiomics features from each ROI, totaling 6138 features. Since the segmentation of the ROIs is based on the atlas, the shape-related features of the ROIs were removed. All radiomics features were based on the following six types: First Order Features; Gray Level Co-occurrence Matrix (GLCM); Gray Level Run Length Matrix (GLRLM); Gray Level Size Zone Matrix (GLSZM); Neighboring Gray Tone Difference Matrix (NGTDM); Gray Level Dependence Matrix (GLDM); (Figure 3b, Figure S1).

Before feature selection and model construction, the two groups were divided into the training set and testing set with a ratio of 7:3; in the PD control group, training set *n* = 35, testing set *n* = 14; in the PD LID group, training set *n* = 37, testing set *n* = 17. The testing set did not participate in the feature selection and classifier-building process, and was only used for model validation. We selected radiomics features extracted from the ROIs. The aim of feature selection was to obtain better classification performance. Firstly, the Levene test was used to determine whether the data conform to the normal distribution. We used the *t*-test to select features with significant differences (*p* < 0.05). To further screen for more recognizable features, we used the least absolute shrinkage and selection operator (LASSO). Next, we calculated the weight and the Pearson correlation coefficient of each selected feature (Figure 3c). 

### 2.5. Classifiers Construction and Validation

Support vector machine (SVM), random forest (RF), and AdaBoost were used to build the classification models. Machine learning was performed on the training set. The testing set was only used for model verification. We performed five-fold cross-validation with 20 repetitions in the training set and validated the testing set to observe the stability of the models. The validation results were based on accuracy, sensitivity, specificity, ROC curves, and AUC of the training and testing set (Figure 3e). The total score of UPDRS III is important for assessing disease progression. Moreover, the progression and duration of the disease are the risk factors of LID. We combined the selected features with the total score of UPDRS III and used the same methods to construct and verify the hybrid models.

## 3. Results

### 3.1. Clinical Information

We compared demographic and some clinical information between the two groups (Table 1). The age, years of education, MoCA score, depression assessment, LEDD, duration of illness before levodopa treatment, REF assessment, the score of the RBD questionnaire, and total score of UPDRS III of the two groups were compared using the Levene test and ANOVA analysis. The gender of the patients in the two groups was tested using the chi-square test. There were no significant differences in these items between the two groups (*p* > 0.05). In the LID group, the duration of levodopa therapy before LID was 3.8 ± 1.7 (mean ± SD) years (Table 1).

### 3.2. Feature Extraction and Selection

We extracted 1023 features from each ROI. A total of 6138 radiomics features were extracted from each sample. We used the Levene test and *t*-test and filtered out 135 features with significant differences; further, we used LASSO (Figure S2) to pick out 36 radiomics features. Next, we performed Pearson correlation analysis and weight analysis for 36 features and removed features with lower weights from two features with correlation coefficients greater than 0.8 (Figures S3 and S4). Finally, we selected 33 features from all radiomics features (Table 2).

### 3.3. Model Validation

**Figure 4:**
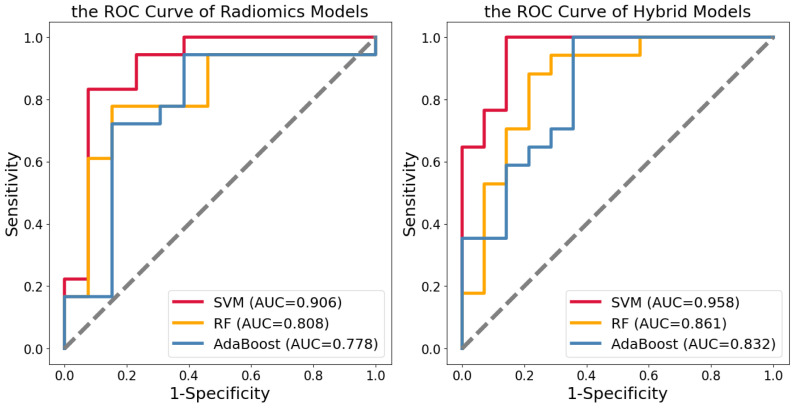
The ROC curves of the radiomics models and hybrid models in the testing set.

After feature selection, we used SVM, RF, and AdaBoost to construct radiomics and hybrid models. The machine learning was only performed on the training set. Validation of the testing set showed SVM had a better classification effect. The hybrid models displayed a better identification effect than the radiomics models. The ROC curves of the testing set for two kinds of models were obtained (Figure 4). In the radiomics model, the AUCs of SVM, RF, and AdaBoost, respectively, were 0.905, 0.808, and 0.778, the sensitivities were 0.778, 0.722, and 0.722, the specificities were 0.923, 0.769, and 0.692, and the accuracies were 0.839, 0.742, and 0.710. In the hybrid models, the AUCs of SVM, RF, and AdaBoost classifiers, respectively, were 0.958, 0.861, and 0.832, the sensitivities were 0.882, 0.882, and 0.765, the specificities were 0.928, 0.714, and 0.786, and the accuracies were 0.903, 0.806, and 0.774 (Table 3). We performed five-fold cross-validation on the training set, validated it in the testing set, and repeated it 20 times. The hybrid model constructed by SVM showed the best performance in identifying LID.

## 4. Discussion

Our research developed a machine learning approach based on radiomics to detect the early onset of LID. This method used T1-weighted MRI, radiomics features of different brain regions, and clinical data. First, we constructed pure radiomics models. Then, the total score of UPDRS III was added to the radiomics features to build hybrid models. The hybrid models performed better in machine learning methods. SVM had better classification and predictive performance than the other two machine learning methods. Compared with traditional clinical evaluation methods used to predict the occurrence of LID, machine learning methods can obtain more accurate predictions in a short time. This is the first study based on radiomics signatures to predict motor complications of PD using machine learning. The hybrid machine learning model had a good effect on predicting LID. This study may provide some new insights into the diagnosis of LID.

In previous studies, radiomics was used to identify the progression of PD. The white matter of the T1-weighted MRI of rapidly progressive PD and slowly progressive PD in 3 years was segmented as ROI, and a classification model was constructed to predict the progression of PD; the AUC of the model was 0.836 and the accuracy was 0.854 [22]. In another study, researchers segmented 19 brain regions from T1-weighted MRI of idiopathic PD, multiple system atrophy, and progressive supranuclear palsy, and constructed radiomics models to diagnose the three diseases; the model accuracy rate was 0.87 [38]. Deep learning models based on DaTscan images could diagnose PD [39]. The clinical image changes of different diseases are based on the pathological basis of the disease. The differences in the radiomics may be more obvious than intuitive medical images. In our study, a single brain region may not be enough to classify PD and PD with LID. The differentiated signatures from several brain regions may help to construct a better classification model.

At present, dopamine replacement therapy is still the main treatment to improve the movement symptoms of patients [40]. Its drawback is that it can cause motor complications such as LID and motor fluctuations. The reasons for the onset of LID are very complicated [41,42]. LID reduces the life quality of PD patients and has significant clinical heterogeneity [43]. Some patients evolved LID in the early stage of dopamine replacement therapy. During the first six years of dopamine placement therapy, about one-third of patients develop LID. The traditional approach to predicting whether a patient will develop early LID is to assess the disease progression and risk factors. This method often takes a long time and results in imprecise conclusions. It is easy to ignore the possibility of early development of LID in patients without risk factors for LID. In our study, the machine learning model based on radiomics uses easily available T1-weighted MRI data. The model obtained the prediction result in a short time and had a high AUC (0.958) and accuracy (0.903) for predicting early LID occurrence. Our work provided some evidence for the potential of machine learning methods to evaluate the occurrence of LID.

The pathogenesis of LID is currently unclear. Studies to date have shown that it may relate to periodic stimulation of dopamine receptors, non-physiological levodopa conversion of serotonergic neurons [44], overactivity of glutamergic transmission in the cortical striatum, and overstimulation of nicotinic acetylcholine receptors on the axons of dopamine release [7]. The pathophysiology of LID is mainly related to changes in the direct and indirect pathways of the cortical basal ganglia thalamic circuit, which regulates normal motor function [26]. A recent study reported significant differences between LID and non-LID patients in the SN of multimodal images. LID had greater neurodegeneration in the SN and altered nigrostriatal connectivity [29]. In our study, radiomics features extracted from SN seem to play a more important role in identifying early LID. An fMRI study on PD showed that executive dysfunctions of the cingulate cortex had a relevant role in dyskinesias-reduced self-awareness [45]. In future research, more attention should be paid to the function of the SN and striatum, and the connection between the striatum and other brain regions in the progression of LID.

For patients at high risk of developing LID, more conservative dopamine replacement therapy should be considered. Doctors can take some early intervention methods to prolong the positive period of dopamine therapy and improve the life quality of these patients. The following therapeutic strategies need to be anticipated: (1) the use of levodopa controlled-release preparations, which contribute to achieving continuous dopaminergic stimulation [24]; (2) the combined use of catechol-O-methyl transferase inhibitors; in a study using a rat PD model, compared with levodopa monotherapy, the combination therapy of levodopa and entacapone effectively reduced all types of LID [46]; and (3) the use of dopamine receptor agonists; a study of a monkey PD model reported the potential of bromocriptine to prevent LID [47]. With the progress of medical research, it is believed that more prevention strategies will be reported, for example, different types of neuroprotective agents and motor rehabilitation training.

Age at onset is an important risk factor for levodopa-induced LID [48]. Studies have found an increased incidence of LID in younger PD patients treated with levodopa [31]. Patients in the later stages of PD have a higher incidence of LID after receiving levodopa therapy [24]. We excluded differences in age and the duration of illness in our present study. In our future study, using the radiomics method to predict the onset age of LID should be considered. Moreover, patients with a longer duration of LID have a poorer quality of life. The duration of LID could be classified and predicted with the radiomics method, which makes sense for a better understanding of LID. Furthermore, the severity of LID is closely related to the life quality of PD. The use of radiomics to classify and predict the severity of LID is also worth considering. In part of UPDRS VI, there are general descriptions of the morning muscle spasms and the severity of disability and pain caused by LID. However, the Independent Spanish Validation of the Unified LID Rating Scale has a detailed description of speech, diet, daily activities, emotions, socialization, dystonia, pain, etc., which are caused by LID [49]. We could also include more LID-related assessments and pay more attention to the correlation between radiomics and the severity of LID in the future.

There are also some limitations in our study. In the process of grouping control patients, we included PD patients who were followed up for more than 6 years and did not develop LID throughout the follow-up period; however, in the subsequent follow-up sessions, patients in the control group still have the possibility of developing LID. Long-term follow-up and observation are required. In the validation results of our study, the hybrid model of SVM had high sensitivity in predicting early LID, but the specificity was not ideal. This indicates that some patients identified as early LID patients may not experience early LID. Therefore, it is necessary to expand the sample size to obtain better machine learning model validation. In addition, external participants need to be added to further studies to validate the model effect. LID is a serious complication related to dopamine replacement therapy, but dopamine therapy still plays a very important role in the treatment of PD [50]. Predicting the occurrence of early LID in PD cannot avoid the need for dopamine replacement therapy. However, for patients identified as suffering from early LID, it is necessary to pay more attention to prevention strategies for LID [51].

## 5. Conclusions

In this study, radiomics and hybrid models based on T1-weighted MRI were constructed to predict the occurrence of early LID in PD. This study extended the role of radiomics in the diagnosis and prognosis of LID. It suggested that radiomics methods have potential for the classification and prediction of PD-related subtypes.

## Figures and Tables

**Table 1:** Demographic and clinical information of the patients.

Statistical Items (Mean ± SD)	PD Control (*n* = 49)	PD with LID (*n* = 54)	*p*-Value
age	65.81 ± 8.51	65.27 ± 9.46	0.762
Edu	12.14 ± 6.54	13.41 ± 6.17	0.315
Sex	33/16	34/20	0.641
MoCA	26.61 ± 3.64	27.04 ± 4.30	0.592
DP	5.69 ± 1.62	5.93 ± 1.80	0.495
H-Y stage	1.71 ± 0.54	1.70 ± 0.5	0.918
duration of PD before levodopa therapy	2.14 ± 1.73	1.57 ± 1.73	0.099
duration of levodopa therapy before LID	-	3.8 ± 1.7	-
LEDD (mg)	167.92 ± 128.12	175 ± 128.35	0.754
REF assessment	6.10 ± 3.52	7.15 ± 2.82	0.098
RBD score	5.61 ± 3.26	5.57 ± 2.97	0.951
UPDRS III	23.37 ± 8.29	22.48 ± 10.04	0.628

Data were displayed as means and SDs. The *p*-values were the results of the ANOVA analysis, *p* > 0.05. SD, standard deviation; sex, male/female; edu, years of education; MoCA, Montreal Cognitive Assessment; DP, Geriatric Depression Scale score; H-Y, Hoehn-Yahr; LEDD, levodopa equivalent daily dose; REF, reflexes assessment; RBD score, RBD questionnaire score; UPDRS III, the third part of Unified Parkinson’s Disease Scale.

**Table 2:** Comparison of the 33 selected radiomics features in the two groups.

Features (Mean ± SD)	PD Control	PD LID	ANOVA (*p*-Value)	Weight
CAU_wavelet-HLH_glszm_HGLZE	6.437 ± 2.362	5.510 ± 1.376	0.0155	−0.0187
PAL_log-sigma-5-0-mm-3D_glcm_Idmn	0.993 ± 0.182	0.994 ± 0.113	0.0498	0.0306
PAL_wavelet-HLL_glszm_HGLZE	12.753 ± 4.513	11.164 ± 3.593	0.0497	−0.0018
PAL_wavelet-HHL_glcm_ClusterShade	0.445 ± 0.625	0.173 ± 0.470	0.0138	0.0743
PAL_wavelet-HHL_gldm_DE	5.402 ± 0.398	5.241 ± 0.344	0.0297	−0.1143
PAL_wavelet-LLL_firstorder_Skewness	0.997 ± 0.301	0.868 ± 0.283	0.0283	0.0426
PAL_wavelet-LLL_glcm_Correlation	0.803 ± 0.201	0.813 ± 0.261	0.0388	0.0303
PUT_wavelet-LLH_glszm_SAE	0.425 ± 0.458	0.441 ± 0.331	0.0472	0.0087
PUT_wavelet-LHL_glszm_LAHGLE	2.02 × 10^7^ ± 6.27 × 10^6^	2.31 × 10^7^ ± 7.15 × 10^6^	0.0332	0.0234
PUT_wavelet-LHH_glszm_SAE	0.603 ± 0.656	0.576 ± 0.523	0.0218	−0.0368
PUT_wavelet-HLL_firstorder_Kurtosis	5.736 ± 0.839	5.316 ± 0.741	0.0081	−0.0546
PUT_wavelet-HHH_glszm_HGLZE	2.602 ± 0.239	2.495 ± 0.295	0.0480	−0.0328
PUT_wavelet-LLL_glcm_Correlation	0.876 ± 0.189	0.883 ± 0.177	0.0380	0.0354
SNpc_wavelet-LHH_firstorder_Skewness	0.774 ± 0.579	0.178 ± 0.465	0.0145	−0.0597
SNpc_wavelet-LHH_ngtdm_Busyness	65.723 ± 36.200	81.908 ± 30.580	0.0120	0.0378
SNpc_wavelet-HHH_glcm_InverseVariance	0.498 ± 0.121	0.493 ± 0.128	0.0404	−0.0324
SNpr_original_firstorder_Minimum	92.899 ± 18.883	80.306 ± 19.247	0.0011	−0.0186
SNpr_original_glszm_SALGLE	0.250 ± 0.100	0.213 ± 0.847	0.0465	−0.0823
SNpr_log-sigma-3-0-mm-3D_glszm_GLNU	5.498 ± 0.904	4.957 ± 0.799	0.0017	−0.0134
SNpr_log-sigma-3-0-mm-3D_glszm_SAE	0.318 ± 0.613	0.284 ± 0.722	0.0114	−0.0391
SNpr_wavelet-LHH_firstorder_Skewness	0.303 ± 0.389	0.118 ± 0.385	0.0175	−0.0581
SNpr_wavelet-HHL_firstorder_90Percentile	6.529 ± 1.476	6.160 ± 1.674	0.0445	0.0085
SNpr_wavelet-HHL_glcm_InverseVariance	0.445 ± 0.156	0.439 ± 0.150	0.0375	−0.0183
SNpr_wavelet-HHL_glszm_LALGLE	1200.777 ± 649.864	1555.148 ± 610.757	0.0052	0.0591
SNpr_wavelet-HHL_glszm_ZoneVariance	2245.971 ± 477.783	2445.259 ± 520.106	0.0462	0.0077
SNpr_wavelet-HHL_gldm_SDE	0.521 ± 0.350	0.499 ± 0.359	0.0021	−0.0264
VTA_wavelet-LHL_ngtdm_Strength	0.319 ± 0.277	0.228 ± 0.159	0.0428	−0.0154
VTA_wavelet-HHL_glcm_Idn	0.840 ± 0.141	0.846 ± 0.163	0.0498	0.0509
VTA_wavelet-HHL_glrlm_GLNUN	0.505 ± 0.999	0.510 ± 0.134	0.0300	0.0190
VTA_wavelet-HHL_glszm_SALGLE	0.137 ± 0.925	0.176 ± 0.103	0.0455	0.0679
VTA_wavelet-HHH_glcm_ClusterProminence	0.431 ± 0.478	0.456 ± 0.401	0.0046	0.0089
VTA_wavelet-HHH_glcm_JointEntropy	1.844 ± 0.805	1.871 ± 0.548	0.0474	0.0366
VTA_wavelet-LLL_ngtdm_Contrast	0.528 ± 0.185	0.685 ± 0.484	0.0356	0.0597

The means and SDs of selected features. The ANOVA analysis showed significant differences in the two groups, *p* < 0.05. The weight of features is displayed. Caudate nucleus (CAU), putamen (PUT), globus pallidus (PAL), SN pars compacta (SNpc), SN reticularis (SNpr), ventral tegmental area (VTA). The abbreviation of feature names can be found in the abbreviation table.

**Table 3:** The model validation of classifiers.

Model	Method	Specificity	Sensitivity	Accuracy	AUC
radiomics model	SVM	0.923	0.778	0.839	0.905
	Random Forest	0.769	0.722	0.742	0.808
	AdaBoost	0.692	0.722	0.710	0.778
hybrid models	SVM	0.928	0.882	0.903	0.958
	Random Forest	0.714	0.882	0.806	0.861
	AdaBoost	0.786	0.765	0.774	0.832

The specificity, sensitivity, accuracy, and AUC of each model. SVM, support vector machine; AUC, the areas under the receiver operating characteristic curve.

## Data Availability

All data and Python code used in this study are available from the corresponding author.

## Supplementary Materials

The following supporting information can be downloaded at: https://www.mdpi.com/article/10.3390/diagnostics13152511/s1, Figure S1: The types of radiomics features that pyradiomics contains; Figure S2: LASSO regression and variable filtering; Figure S3: Weights of the 33 selected radiomics features; Figure S4: Pearson correlation coefficient heatmap of 33 selected radiomics features; the table of abbreviations.
