# Supplementary material for: Radiomics and Hybrid Models Based on Machine Learning to Predict Levodopa-Induced Dyskinesia of Parkinson’s Disease in the First 6 Years of Levodopa Treatment
Source: Diagnostics (Basel). 2023 Jul 27;13(15):2511. doi: 10.3390/diagnostics13152511 (PMC10417024; doi:10.3390/diagnostics13152511)
Supplement: Supplementary file 1 [file diagnostics-13-02511-s001.zip › Abbreviations.pdf]

Abbreviations	
Advanced Normalization Tools	ANTs
Area under the ROC curve	AUC
Arterial spin labeling	ASL
Caudate nucleus	CAU
Dependence Entropy	DE
Diffusion-weighted imaging	DWI
FMRIB Software Library	FSL
Globus pallidus	PAL
Gray Level Co-occurrence Matrix	GLCM
Gray Level Dependence Matrix	GLDM
Gray Level NonUniformity	GLNU
Gray Level NonUniformity Normalized	GLNUN
Gray Level Run Length Matrix	GLRLM
Gray Level Size Zone Matrix	GLSZM
High Gray Level Zone Emphasis	HGLZE
Informational Measure of Correlation 1	IMC1
Inverse difference normalized	IDN
Large Area High Gray Level Emphasis	LAHGLE
LargeAreaLowGrayLevelEmphasis	LALGLE
Levodopa equivalent dose	LEDD
Levodopa-induced dyskinesia	LID
Magnetic resonance imaging	MRI
Montreal cognitive assessment	MoCA
Neighbouring Gray Tone Difference Matrix	NGTDM
Parkinson's disease	PD
Putamen	PUT
Random forest	RF
Rapid eye movement sleep disorder	RBD
Receiver operating characteristic	ROC
Reflexes assessment	REF
Regions of interest	ROIs
Size Zone Non Uniformity	SZNN
Small Area Emphasis	SAE
Small Area Low Gray Level Emphasis	SALGLE
Small Dependence Emphasis	SDE
Substantia nigra	SN
Substantia nigra pars compacta	SNpc
Substantia nigra reticularis	SNpr
Support vector machine	SVM
Susceptibility imaging	SWI
The least absolute shrinkage and selection operator	LASSO
Unified Parkinson's Disease Rating Scale part III	UPDRS III
Ventral tegmental area	VTA
