# Supplementary figures and images for: Radiomics and Hybrid Models Based on Machine Learning to Predict Levodopa-Induced Dyskinesia of Parkinson’s Disease in the First 6 Years of Levodopa Treatment
Source: Diagnostics (Basel). 2023 Jul 27;13(15):2511. doi: 10.3390/diagnostics13152511 (PMC10417024; doi:10.3390/diagnostics13152511)

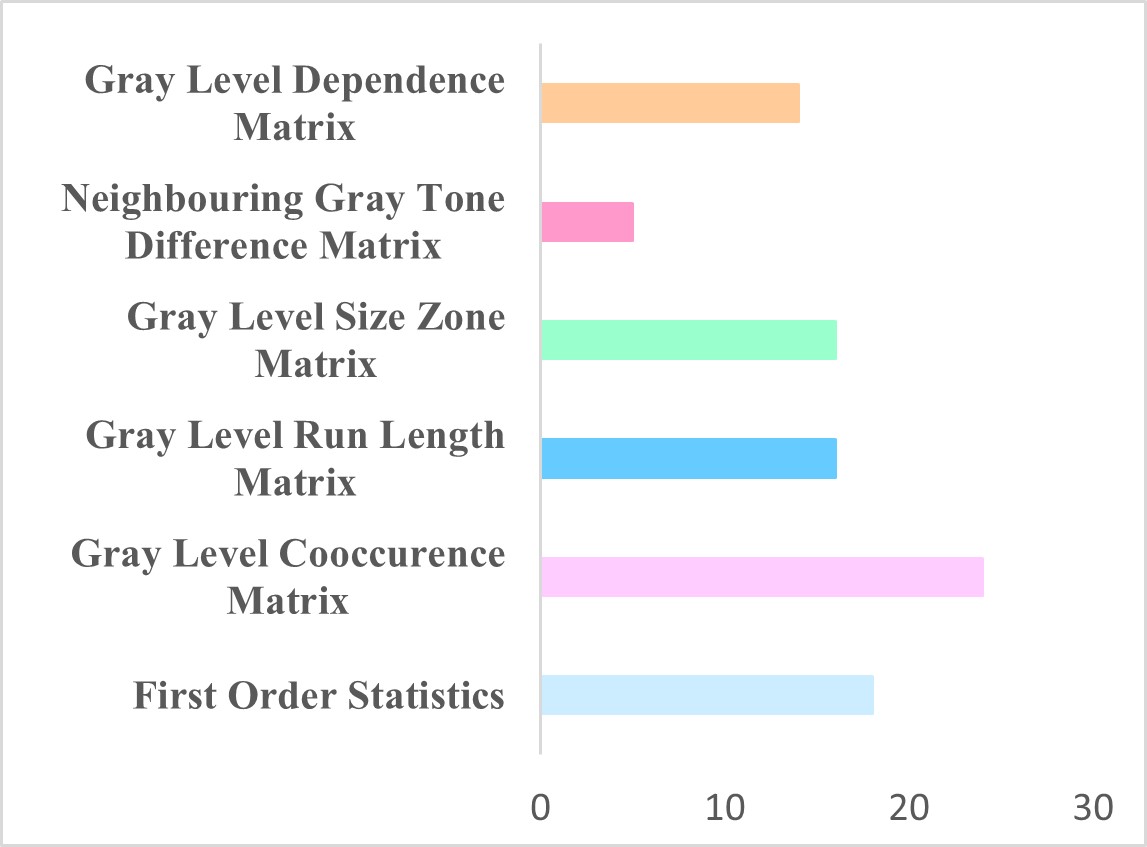

Supplement: Supplementary file 1 [file diagnostics-13-02511-s001.zip › Figure S1 The types of radiomics features that pyradiomics contains.jpg]

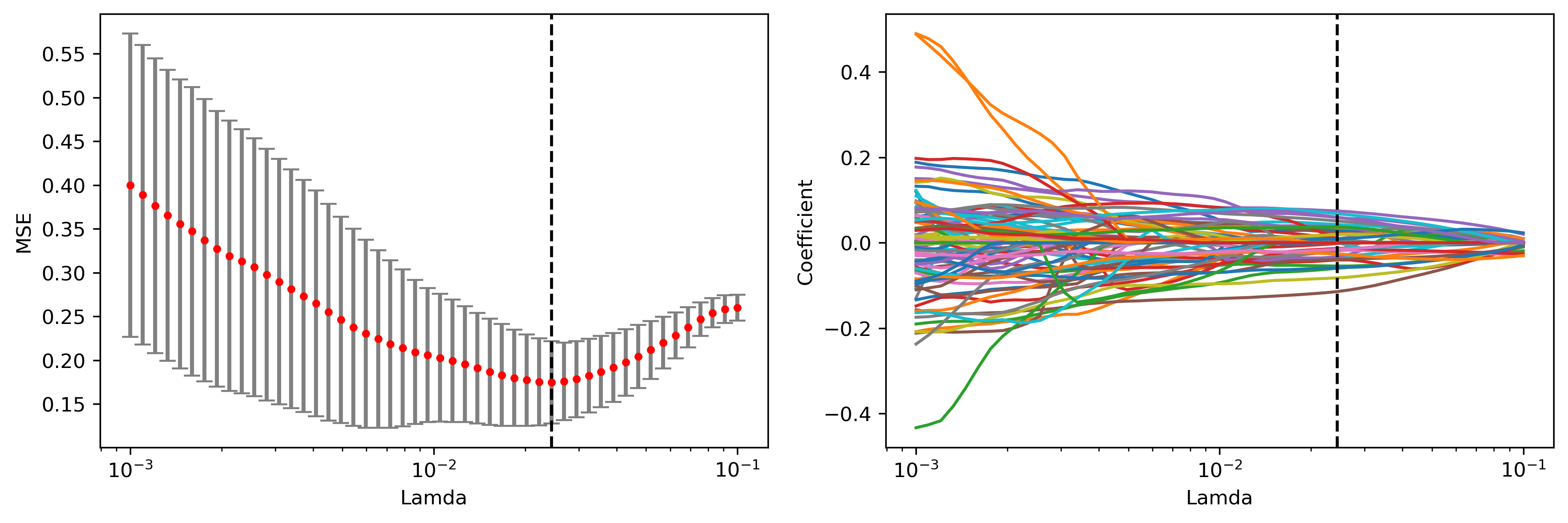

Supplement: Supplementary file 1 [file diagnostics-13-02511-s001.zip › Figure S2 LASSO regression and variable filtering.tif]

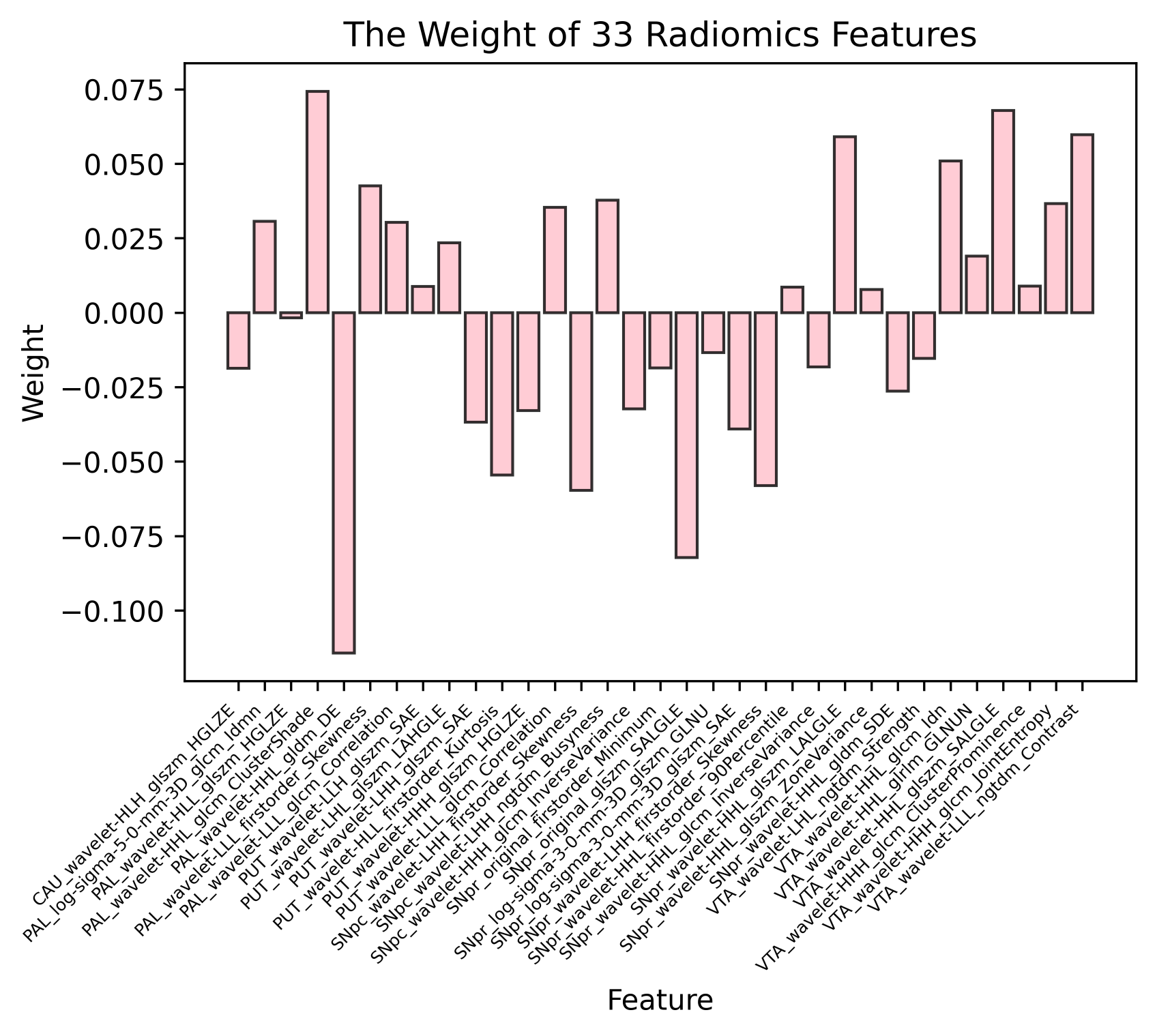

Supplement: Supplementary file 1 [file diagnostics-13-02511-s001.zip › FigureS3Weights of the 33 selected radiomics features.tif]

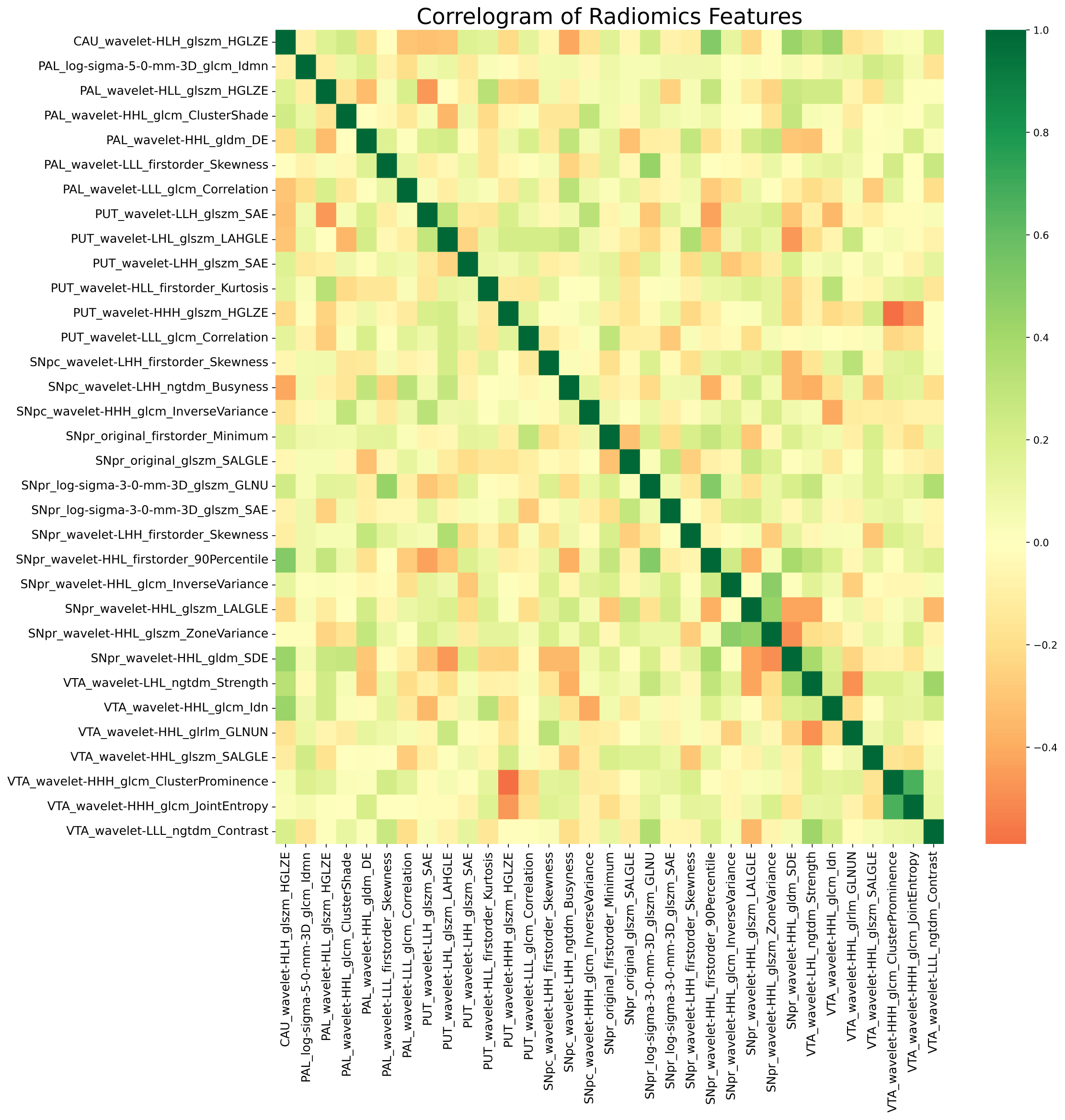

Supplement: Supplementary file 1 [file diagnostics-13-02511-s001.zip › FigureS4Pearson correlation coefficient heatmap of 33 selected radiomics features.tif]
